# Prevalence of high-risk human papillomavirus types among women aged 30–49 years in the Chuy region, Kyrgyzstan

**DOI:** 10.1186/s13027-025-00727-2

**Published:** 2026-01-02

**Authors:** Turatbek Kozukeev, Olga EM, Iza Ciglenecki, Evelyne Wachira, Asel Tenizbaeva, Adrian Guadarrama, Masha Pastrana, Manuel Albela, Jean Kalibushi

**Affiliations:** 1 Médecins Sans Frontières, Bishkek, Kyrgyzstan; 2 National Institute of Public Health, Ministry of Health, Bishkek, Kyrgyzstan; 3 Médecins Sans Frontières, Geneva, Switzerland; 4 Family Medical Center #6, Ministry of Health, Bishkek, Kyrgyzstan; 5 Médecins Sans Frontières, Berlin, Germany

## Abstract

**Introduction:**

Human papillomavirus (HPV) is a leading cause of cervical cancer globally. High-risk (HR) HPV types HPV16 and 18 are responsible for 70% of cases. However, limited data exist for Kyrgyzstan, where cervical cancer is the second most common cancer in women. This study aimed to establish a baseline HR-HPV prevalence, genotype distribution as well as risk factors for HR-HPV persistence among women aged 30–49 in the Chuy region, before the national HPV vaccination program was fully implemented.

**Methods:**

A cross-sectional study was conducted between January and May 2024 in two facilities in rural Sokuluk District and one in Bishkek City. Cervical specimens from 1213 women were tested using the Xpert HPV assay, which detects oncogenic HPV types 16, 18/45 and other combined types (31, 33, 35, 52 and 58 are combined as P3, 51 and 59 as P4 and 39, 56, 66 and 68 as P5). Visual inspection with acetic acid (VIA) was performed, and demographic, social, and behavioral data were collected through structured questionnaires. Descriptive and logistic regression analyses were conducted to explore HPV prevalence and associated risk factors.

**Results:**

The overall HR-HPV prevalence was 9.0% (109/1213, 95% CI 7.5–10.7%). HR-HPV prevalence was higher in Sokuluk (56/570, 9.8%) compared to Bishkek (33/424, 7.8%). HPV16 was the most prevalent genotype (2.5%), followed by pooled types P3 (2.3%) and P5 (2.1%). Among women with detected HPV, 13.6% had a positive VIA result. Alcohol consumption was significantly associated with HR-HPV detection (adjusted odds ratio (aOR) 1.81, 1.14–2.82, *p* = 0.010), while previous cervical pathology was protective factor (aOR 0.63, 0.41–0.95, *p* = 0.032).

**Conclusions:**

This study represents the first comprehensive data on the prevalence and distribution of HR-HPV in Kyrgyzstan. The findings emphasize the importance of tailored public health interventions, including expanded HPV vaccination, and scaling-up HPV-testing based cervical cancer screening programs. Further research is needed to evaluate the impact of these interventions on reducing the burden of HPV-associated cervical cancer in Kyrgyzstan.

**Clinical trial number:**

Not applicable.

## Introduction

Human papillomavirus (HPV) is a widespread sexually transmitted infection, with over 200 known types, of which at least 14 are classified as high-risk or high oncogenic risk due to their strong association with cervical cancer [1]. Cervical cancer is the fourth most common cancer among women worldwide and the second most common cancer among women aged 15–44 years [2, 3]. In 2020, an estimated 604,000 new cases and 342,000 deaths from cervical cancer occurred worldwide, with more than 85% of these deaths occurring in low-and middle-income countries (LMICs) [4]. Among the high risk oncogenic HPV (HR-HPV) types, HPV 16 and 18 are particularly concerning, accounting for approximately 70% of cervical cancer cases globally [5]. In a meta-analysis of HPV type distribution in women with cervical cancer across different regions, HPV 16 was the most prevalent type in all regions, followed by HPV 18, 31, 33, and 45 [6]. Studies conducted in Central Asia showed similar distribution; HPV 16 was by far the most common HPV type in Uzbekistan [7] and Kazakhstan [8, 9].

Cervical cancer screening programs, including HPV DNA testing, have been widely implemented in many high-income countries and have proven to be effective in reducing cervical cancer incidence [10, 11]. Similarly, the introduction of HPV vaccine in routine immunization schedules led to a significant reduction in the prevalence of vaccine-type HPV infections and a corresponding decrease in the incidence of cervical precancerous lesions among vaccinated populations [12]. Studies using cancer registers have shown near-elimination of cervical cancer in women vaccinated at age of 12–13 years [13].

However, despite the advances in prevention, screening and early treatment of cervical cancer, resource-limited countries face numerous challenges in implementing programs. In Kyrgyzstan, cervical cancer is the second most common cancer among women, with an estimated age-standardized incidence rate of 14.1 per 100,000 women and mortality of 11.1 per 100’000 women in 2022 [14]. The HPV vaccination was only introduced in 2022, targeting girls aged 11 years [15]. In 2023, the Ministry of Health issued an updated clinical guidelines for screening and treatment of pre-cancerous cervical lesions [16], aligning the recommendations with current WHO guidelines [17]. The guideline recommends HPV testing or cytology as the screening test for all women aged 30–59 years old, replacing visual inspection with acetic acid (VIA) as the screening method [15]. Screening should be available at the primary healthcare level and free of charge. However, in practice the new guideline is implemented as pilot projects and screening tests are not universally available in routine settings and women are sent to private laboratories at their cost (personal communication). Similarly, the implementation of the previous guideline was limited; in 2021 the WHO estimated that only 32% of women aged 30–49 had been screened for cervical cancer within the past five years [18].

Médecins Sans Frontières (MSF), an international humanitarian non-governmental organization, supported the Ministry of Health (MOH) of Sokuluk district in Chuy region, in introducing cervical and breast cancer screening at two primary health facilities. No epidemiological data is available on prevalence of HR-HPV and distribution of genotypes in Kyrgyzstan. This study aims to address this gap by investigating the prevalence of HR-HPV types, genotype distribution as well as risk factors for HR-HPV persistence among women aged 30–49 years in the Chuy region in Kyrgyzstan. The findings will contribute to the understanding of HPV epidemiology in this under-researched region and support efforts to reduce cervical cancer incidence through tailored public health interventions.

## Materials and methods

### Study design and settings

A hospital based cross-sectional study was conducted in three public health facilities in Chuy region from January to May 2024. These health facilities were two primary health care centers in Sokuluk district of Jani-Jer and Sokuluk (predominantly rural), where MSF has been screening women of reproductive age for cervical lesions using the VIA method since 2022, and one urban primary health care center in Bishkek city (FMC#6).

### Study population

Women, aged 30–49 years, attending routine gynecologic or family planning services at 3 study facilities were eligible for the enrollment in the study, if they had no acute severe febrile illness, had not undergone total hysterectomy, were not considered pregnant (as per oral feedback from the women), and had not undergone thermoablation in the last year. Information about cervical cancer screening and the study was provided with the support of MSF health promotion team through the community organizations, health care workers, stakeholders and local media. All women presenting to the study facilities fulfilling the criteria were enrolled in the study if they provided informed consent. A cervical specimen was collected for detection of major HR-HPV types, followed by a VIA test and completion of a structured questionnaire.

### Sample size and sampling technique

The sample size was determined using a single population proportion formula based on an Eastern European HPV prevalence of 14% and a precision of 2% with a 95% confidence interval. This resulted in a total sample size of 1152 for the three selected health facilities where women were recruited consecutively.

### Study procedures

#### Data collection

A structured questionnaire on socio-demographic characteristics and behavioral risk factors for cervical cancer was administered by trained study doctors and nurses. The questionnaire was piloted during the training and minor adjustments were made prior to implementation (pilot data was not included in the analysis).

#### Molecular HPV testing

We used Cepheid’s Xpert^®^ platform and Xpert^®^ HPV cartridge for HPV detection. All laboratory testing was performed in the Sokuluk primary health center. Endocervical swabs were collected by trained medical staff by inserting the swabs into the endocervix and rotating them counterclockwise to a complete circle three times. The collected swab was then placed into the PreservCyt^®^ Solution (Hologic Corp.) with the patient code, and at the end of the working day the collected materials were transported to the MSF screening point in Sokuluk primary health center.

Xpert HPV testing was used for detection of HR-HPV types. This is a qualitative in vitro test to detect the E6/E7 region of the HPV viral DNA genome. The test provides multiplex amplification of target DNA by real-time polymerase chain reaction (PCR) of 14 HR-HPV types in a single analysis. The channels contain primers and probes to detect specific genotypes or pooled results as follows: “HPV16 Primary” for HPV16, “HPV 18_45 Primary” for a pooled HPV 18/45 result, “P3 Primary” for a pooled result of any of HPV types 31, 33, 35, 52, or 58, “P4 Primary” for a pooled result of any of HPV types 51 or 59, and “P5 Primary” for a pooled result of any of HPV types 39, 56, 66, or 68. Detection of HPV types 16, 18/45 and other types in cervical smears was performed using the GXHPV-CE-10 cartridge according to the manufacturer’s instructions. The test results were also interpreted according to the manufacturer’s instructions. Samples where the HPV result was “Invalid” were retested using a new cartridge.

#### Detection of pre-cancerous cervical lesions using VIA

The VIA test was performed after obtaining specimens for HR-HPV types; a 5% acetic acid solution was applied to the cervix with a cotton swab. Within 60 s, a cervical precancerous lesion was seen as whitish area called acetowhite lesion but without that finding, VIA was considered negative. Women with a positive VIA result and the lesion covering less than 75% of the cervix were advised to undergo thermoablation, and those with more than 75% of lesions were referred to national oncological center. VIA was not performed if transformation zone was not visible.

#### Cytology

Liquid-based cytology was performed for participants if VIA was not possible and for those where HPV result was positive and VIA result was negative (results not shown).

### Data analysis

The data collected on the paper questionnaires were checked for completeness before encoding into REDCap software. STATA version 18 and R were used for the analyses. Frequencies, proportions, and summary statistics were used to describe the study population with relevant variables. Logistic regression was used to identify factors associated with HPV infection, and odds ratios with 95% CI were used to estimate the strength of association. To adjust for confounding factors, we included age group as default and variables from the univariate analysis (p-value < 0.05 was considered a statistically significant association) into the multivariate logistic regression model, using backward selection. Missing values were not imputed and were excluded from the logistic regression analysis.

## Results

There were a total of 1286 records in the REDCap database, of which 61 were duplicates, 11 had an HPV result of “Error” or “Invalid” after retesting, and one woman did not meet the study inclusion criteria for age. After excluding duplicates, records with indeterminate HR-HPV test results, and records that did not meet study criteria, 1213 records were included for analysis. Table 1 summarizes the baseline characteristics of the study participants, including the distribution of HR-HPV detection.

**Table 1:** Socio-demographic and behavioral characteristics of enrolled participants overall and those with HR-HPV detected and the univariate risk factor analysis results

Characteristics	Overall *N* = 1,213 *n*(%)	Presence of HR-HPV types *N* = 109 *(9%)* *n*(%)	cOR	95% CI	*p*-value
**Age** (mean ± SD)	39.2 ± 5.4	39.1 ± 5.8			
**Age group**					
45–49	254 (21%)	24 (22%)	—	—	
30–34	285 (23%)	30 (28%)	1.13	0.64, 2.00	0.7
35–39	359 (30%)	29 (27%)	0.84	0.48, 1.49	0.6
40–44	315 (26%)	26 (24%)	0.86	0.48, 1.55	0.6
Location of the study*					
Sokuluk	570 (47%)	56 (51%)	—	—	
Jani-Jer	219 (18%)	20 (18%)	0.92	0.53, 1.55	0.8
Bishkek	424 (35%)	33 (30%)	0.77	0.49, 1.21	0.3
**Nationality**					
Other nationality	82 (6.8%)	11 (10%)	—	—	
Kyrgyz	1,131 (93%)	98 (90%)	0.61	0.33, 1.26	0.2
**Place of permanent residence**					
Bishkek	463 (38%)	38 (35%)	—	—	
Chuy region	750 (62%)	71 (65%)	0.86	0.56, 1.28	0.5
**Marital status**					
Married	1,057 (87%)	84 (77%)	—	—	
Not married**	156 (13%)	25 (23%)	2.21	1.34, 3.53	**0.001**
**Education**					
Higher education	518 (43%)	43 (39%)	—	—	
Primary (1–4 grades)	7 (0.6%)	0 (0%)	0.00		> 0.9
Secondary education	688 (57%)	66 (61%)	1.17	0.79, 1.76	0.4
**Work status**					
Not employed	563 (46%)	41 (38%)	—	—	
Employed	650 (54%)	68 (62%)	1.49	1.00, 2.25	0.055
**Family income per month**					
Less or equal to 55 USD	505 (42%)	41 (38%)	—	—	
Between 56 and 112 USD	325 (27%)	28 (26%)	1.07	0.64, 1.75	0.8
Equal or more than 113 USD	383 (32%)	40 (37%)	1.32	0.83, 2.09	0.2
**Smokes cigarettes**					
No	1,109 (91%)	94 (86%)	—	—	
Yes	103 (8.5%)	15 (14%)	1.84	0.99, 3.22	**0.042**
Unknown	1 (< 0.1%)	0 (0%)			
**Duration of smoking**					
Non-smoker	1,109 (91%)	94 (86%)	—	—	
Smokes since less than 2 years	15 (1.2%)	3 (2.8%)	2.70	0.61, 8.68	0.13
Smokes since more than 2 years	88 (7.3%)	12 (11%)	1.70	0.86, 3.14	0.10
Missing	1 (< 0.1%)	0 (0%)			
**Frequency of smoking per day**					
Non-smoker	1,109 (91%)	94 (86%)	—	—	
1–5 times a day	81 (6.7%)	13 (12%)	2.06	1.06, 3.76	**0.024**
6–10 times a day	16 (1.3%)	1 (0.9%)	0.72	0.04, 3.61	0.8
More than 10 times a day	6 (0.5%)	1 (0.9%)	2.16	0.11, 13.6	0.5
Missing	1 (< 0.1%)	0 (0%)			
**Alcohol consumption**					
None	923 (76%)	69 (63%)	—	—	
Yes	290 (24%)	40 (37%)	1.98	1.30, 2.98	**0.001**
**Age of onset of menstruation**					
14 years or less	690 (57%)	61 (56%)	—	—	
15 years or more	523 (43%)	48 (44%)	1.04	0.70, 1.55	0.8
**Age of first sexual intercourse**					
25 years and older	175 (14%)	13 (12%)	—	—	
18 years and younger	247 (20%)	30 (28%)	1.72	0.89, 3.51	0.12
19–24 years	791 (65%)	66 (61%)	1.13	0.63, 2.20	0.7
**Number of pregnancies**					
No pregnancy	22 (1.8%)	6 (5.5%)	—	—	
1–5 pregnancies	770 (63%)	72 (66%)	0.28	0.11, 0.79	**0.009**
6 and more pregnancies	421 (35%)	31 (28%)	0.21	0.08, 0.62	**0.003**
**Number of children born alive**					
No children	38 (3.1%)	9 (8.3%)	—	—	
1–3 children	685 (56%)	63 (58%)	0.33	0.15, 0.76	**0.006**
4 and more children	490 (40%)	37 (34%)	0.26	0.12, 0.63	**0.001**
**Use contraceptive**					
Uses contraception	538 (44%)	39 (36%)	—	—	
Does not use contraception	675 (56%)	70 (64%)	1.48	0.99, 2.25	0.060
**Previously screened for cervical cancer**					
Previously screened	876 (72%)	82 (75%)	—	—	
Previously not screened	327 (27%)	26 (24%)	0.84	0.52, 1.31	0.4
Unknown	10 (0.8%)	1 (0.9%)			
**Previously diagnosed with cervical pathology**					
No	658 (54%)	71 (65%)	—	—	
Yes	544 (45%)	37 (34%)	0.60	0.39, 0.91	**0.017**
Unknown	11 (0.9%)	1 (0.9%)			
**Previously treated for cervical pathology (thermoablation)**					
No	829 (68%)	84 (77%)	—	—	
Yes	378 (31%)	25 (23%)	0.63	0.39, 0.98	0.050
Unknown	6 (0.5%)	0 (0%)			
**Treated for STIs within the past 5 years**					
No	1,115 (92%)	104 (95%)	—	—	
Yes	94 (7.7%)	5 (4.6%)	0.55	0.19, 1.25	0.2
Unknown	4 (0.3%)	0 (0%)			
**Partner treated for STIs in the last 5 years**					
No	1,111 (92%)	99 (91%)	—	—	
Yes	61 (5.0%)	4 (3.7%)	0.72	0.21, 1.79	0.5
Unknown	41 (3.4%)	6 (5.5%)			
**Number of sexual partners in the last 5 years**					
No partner	22 (1.8%)	4 (3.7%)	—	—	
1 partner	1,168 (96%)	102 (94%)	0.43	0.16, 1.51	0.13
2 and more partners	23 (1.9%)	3 (2.8%)	0.68	0.12, 3.46	0.6
**Previously tested for HIV and the result is known**					
Previously tested and knows HIV result	940 (77%)	84 (77%)	—	—	
Does not know the HIV test result	247 (20%)	21 (19%)	0.95	0.56, 1.53	0.8
Unknown	24 (2.0%)	4 (3.7%)			
Missing	2 (0.2%)	0 (0%)			
**VIA test results**					
Negative	1,079 (89%)	91 (83%)	—	—	
Positive	103 (8.5%)	14 (13%)	1.71	0.90, 3.03	0.082
Not done	31 (2.6%)	4 (3.7%)	1.61	0.47, 4.23	0.4

CI = Confidence Interval, cOR = crude Odds Ratio, HR-HPV – High Risk of Human Papillomavirus; STI– Sexually transmitted infections; HIV – human immunodeficiency virus; VIA = visual inspection with acetic acid

*Sokuluk and Jani-Jer are predominantly rural, Bishkek is urban

** Not Married - includes divorced or widowed people

**Fig. 1:**
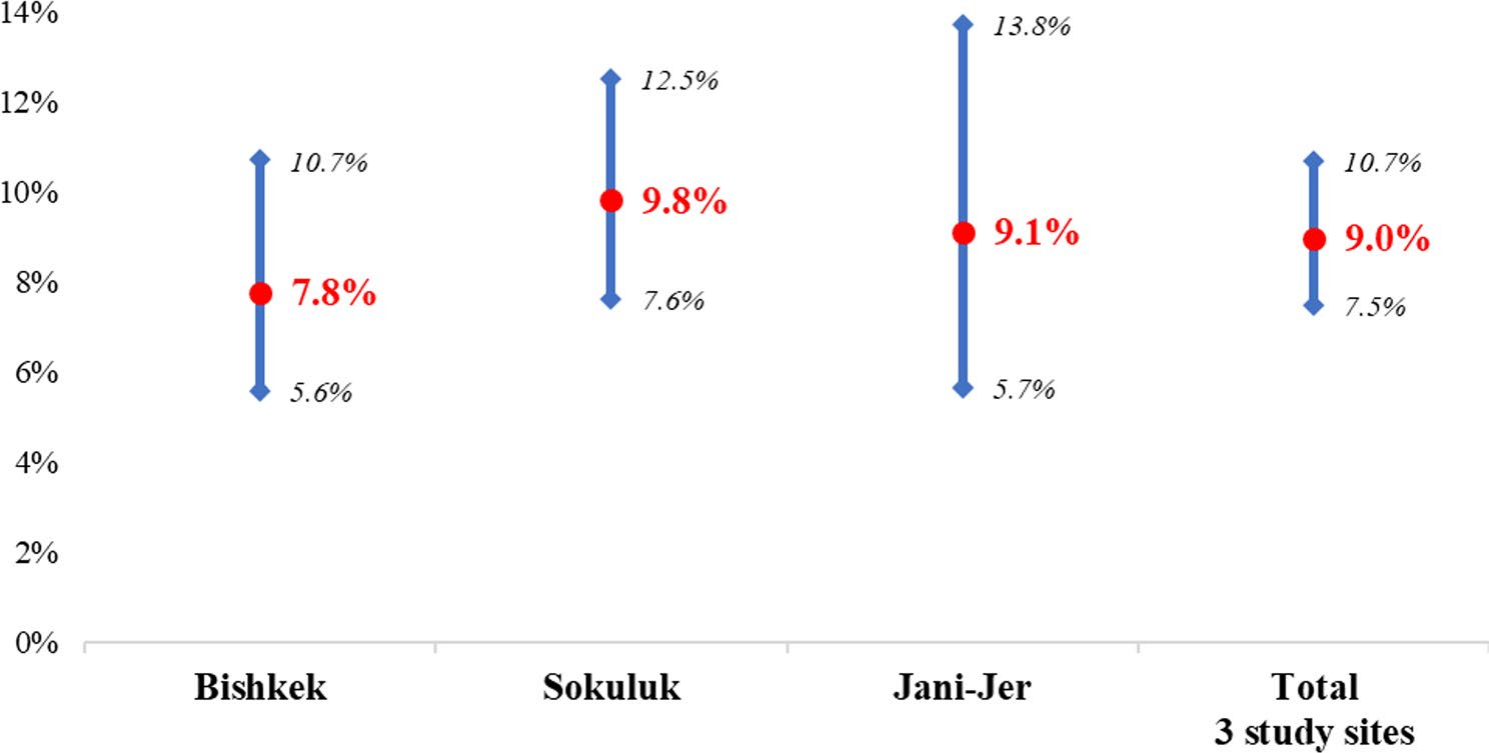
Distribution of high-risk HPV types detected by study sites with 95% confidence intervals (*n* = 1213)

**Fig. 2:**
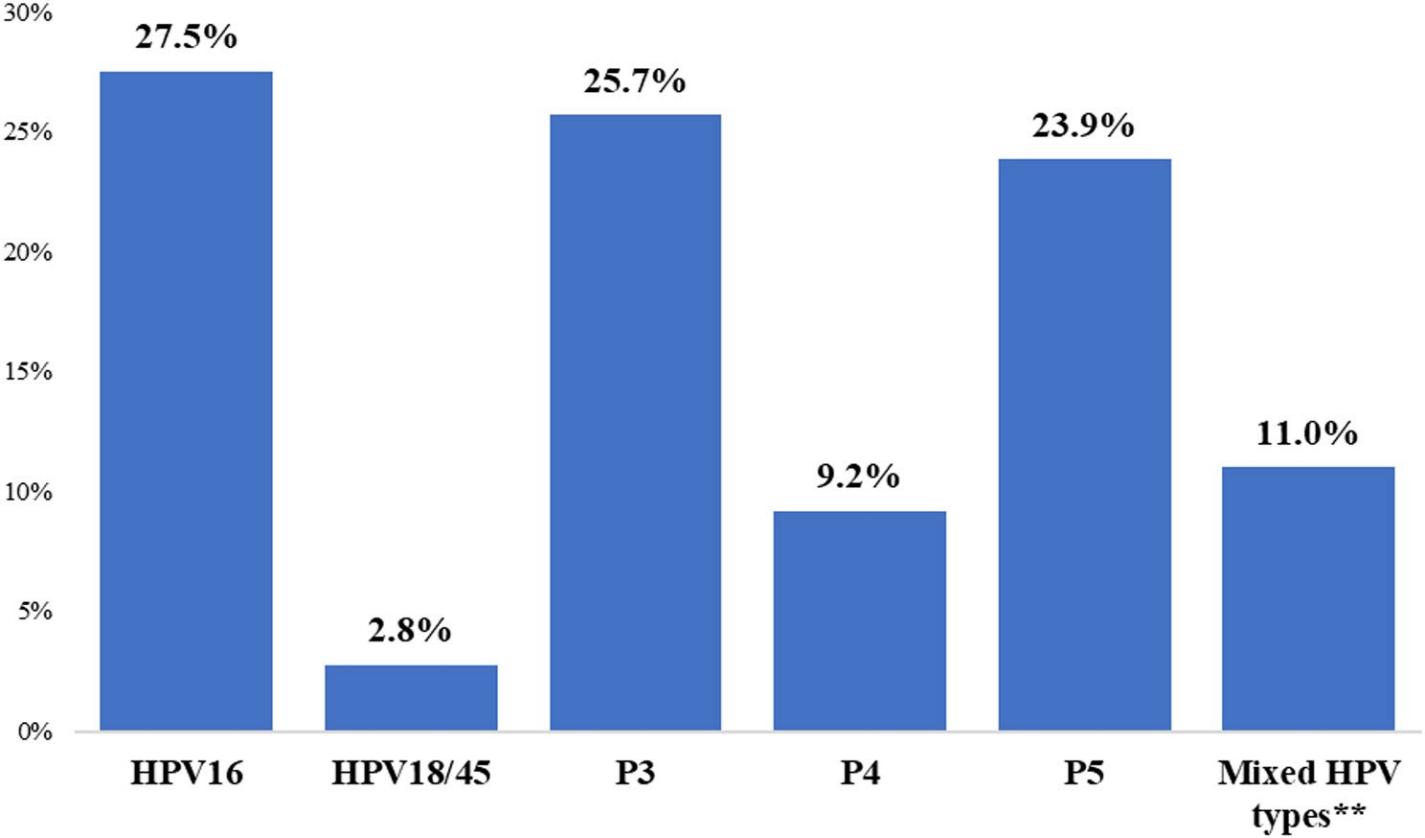
Proportion of HR-HPV type distribution among HPV-positive (*n* = 109)*. Abbreviation: HR HP – High Risk of Human Papillomavirus. The test system used allowed the detection of other HPV types in 3 subgroups, which were grouped as follows: **P3–31**, 33, 35, 52 and 58; **P4–51** and 59; **P5–39**, 56, 66 and 68. *Each infection is counted only once (either as individual type or as part of mixed infection). Mixed HPV types** - there were 7 cases with more than one HPV type detected, they were combined into one group: HPV16 + HPV18/45; HPV16 + P3; HPV16 + P5; HPV18/45 + P3; HPV18/45 + P5; HPV16 + 18/45 + P4 (1 + 2 + 1 + 1 + 1 + 1 + 1 + 1)

The study included 1213 women aged 30–49 years, with a mean age of 39.2 years (± SD = 5.4). 570 (47%) were recruited in Sokuluk, 219 (18%) in Jani-Jer and the remaining 424 (35%) in Bishkek. Majority (1131, 93.2%) were of Kyrgyz ethnicity, 695 (57.3%) had secondary education, 563 (46.4%) were unemployed and majority (1057, 87.1%) were married. Few women (103, 8.5%) reported to smoke cigarettes and less than a quarter (290, 24.0%) reported alcohol consumption. The average per capita family income per month reported for 505 (41.6%)of respondents was less than $55, for 325 (26.8%) between $56 and $112, and for 383 (31.6%) more than $112. 247 (20.4%) women had their first sexual intercourse at the age of 18 years and younger, 38 (3.1%) had no children, and 490 (40.4%)had 4 or more children. Contraception was used by 538 (44.4%) of women. 124 (10.2%) women reported to have had two or more sexual partners. VIA identified only 14 (13%) of HR-HPV-positive women. The overall prevalence of HR-HPV in the study population was 9.0% (95% CI 7.5–10.7), with a slightly higher prevalence in Sokuluk (9.8%) and Jani-Jer (9.1%) compared to Bishkek (7.8%), but these differences were not statistically significant (*p* = 0.53) (Table 1; Fig. 1). Table 2 shows the prevalence of HR-HPV types among all tested women; the most common HR-HPV types was HPV 16 at 2.5%, other HPV types (P3) at 2.3% including HPV types 31, 33, 35, 52 or 58, and other HPV types (P5) at 2.1% including types 39, 56, 66 or 68. Among 109 HPV positive samples, HPV 16 represented 27.5%, P3 25.7% and P5 23.9% (Fig. 2).

**Table 2:** Prevalence of high-risk HPV types (*n* = 1213)*

HR HPV types	Number (%)	95% CI
HPV16	30 (2.5)	1.74–3.51
HPV18/45	3 (0.2)	0.08–0.72
HPV-Other (P3)	28 (2.3)	1.6–3.32
HPV-Other (P4)	10 (0.8)	0.45–1.51
HPV-Other (P5)	26 (2.1)	1.47–3.12
Mixed HPV (P3 + P5)	3 (0.2)	0.08–0.72
Mixed HPV (P4 + P5)	1 (0.1)	0.01–0.47
Mixed HPV (P3 + P4 + P5)	1 (0.1)	0.01–0.47
Mixed HPV16 + 18/45	1 (0.1)	0.01–0.47
Mixed HPV16 + P3	2 (0.2)	0.05–0.60
Mixed HPV16 + P5	1 (0.1)	0.01–0.47
Mixed HPV18/45 + P3	1 (0.1)	0.01–0.47
Mixed HPV18/45 + P5	1 (0.1)	0.01–0.47
Mixed HPV16 + 18/45 + P4	1 (0.1)	0.01–0.47

HR HPV – High Risk Human Papillomavirus; CI – Confidence Interval

The test system used allowed the detection of other HPV types in 3 subgroups, which were grouped as follows: **P3–31**, 33, 35, 52 and 58, **P4–51** and 59 and **P5–39**, 56, 66 and 68﻿

*Each infection is counted only once (either as individual type or as part of mixed infection)﻿

In univariate analysis, the HR-HPV positivity was associated with not being married (crude odds ratio (cOR) 2.21 (95%CI 1.3–3.5, *p* < 0.001), alcohol consumption (cOR 1.98, 95%CI 1.3–2.9, *p* < 0.001), cigarette smoking cOR (1.89, 95%CI 1.0–3.3, *p* = 0.042), and smoking 5 or less times per day (cOR 2.14, 95% CI 1.6–3.9, *p* = 0.024). Women with 1–5 pregnancies (cOR 0.27,95%CI: 0.1–0.7, *p* = 0.009) and ≥ 6 pregnancies (cOR 0.26, 95%CI: 0.1–0.5), similarly to women with 1–3 children (cOR 0.32,95%CI 0.1–0.7, *p* = 0.006) and ≥ 4 children (cOR 0.26, 95%CI 0.1–0.6, *p* < 0.001)) had lower odds of having HR-HPV compared to women with no previous pregnancies or children. Women previously diagnosed with cervical pathology also had lower odds of HR-HPV detection (cOR 0.60, 95%CI 0.4–0.9, *p* = 0.017).

**Table 3:** Multivariate logistic regression analysis of the risk factors for HR-HPV infection

Characteristic	aOR	95% CI	*p*-value
Age group			
45–49	—	—	
30–34	1.27	0.70, 2.33	0.4
35–39	0.96	0.53, 1.74	0.9
40–44	0.96	0.53, 1.76	> 0.9
Marital status			
Married	—	—	
Not married	1.61	0.93, 2.70	0.079
Smokes cigarettes			
No	—	—	
Yes	1.06	0.53, 1.99	0.9
Alcohol consumption			
None	—	—	
Yes	1.81	1.14, 2.82	0.010
Number of pregnancies			
No pregnancy	—	—	
1–5 pregnancies	0.43	0.16, 1.30	0.11
6 and more pregnancies	0.36	0.13, 1.14	0.066
Previously diagnosed with cervical pathology			
No	—	—	
Yes	0.63	0.41, 0.95	0.032

Abbreviations: CI = Confidence Interval, OR = Odds Ratio

After adjusting for confounding factors identified in univariate analysis and age (marital status, smoking cigarettes, consuming alcohol, number of pregnancies and previous diagnoses of cervical pathology) (Table 3), only alcohol consumption (adjusted odds ratio (aOR) 1.81, 95%CI 1.14–2.82; *p* = 0.010) was associated with increased odds of HPV infection, while the history of previously diagnosed cervical pathology was associated with a decreased odds of having HPV (OR 0.63, 95%CI 0.41–0.95; *p* = 0.032).

## Discussion

The aim of this study was to investigate the prevalence of HR-HPV infection among 30–49 years old women in Chuy region of Kyrgyzstan and its association with various socio-demographic and behavioral risk factors. The overall prevalence of HR-HPV (9%) is comparable to the average prevalence of HPV infection among women worldwide, which ranges from 5% to 15% depending on the region [7, 9, 19]. Regionally, a study conducted in Uzbekistan between 2021 and 2023 has shown similar prevalence (7.4%) of 12 HR-HPV [7], however studies from Kazakhstan have shown much higher HR-HPV prevalence ranging from 16 to 43% [9]. The higher prevalence of HR-HPV in the two rural sites (Sokuluk and Jani-Jer), although not statistically significant, suggests that local environmental, cultural or health factors may influence the HPV transmission, consistent with findings from studies in Tanzania and Gabon, where regional differences in HPV prevalence were also observed [20, 21].

Notably, HPV type 16 was the most common type (2.5%), consistent with global [19] and regional reports [7–9]. The presence of other HR-HPV types such as HPV 31, HPV 33, HPV 52, and HPV 39 (grouped as HPV-P3 and P5) highlights the complex nature of HPV infection and suggests that broader screening programs should consider a wider range of HPV strains rather than focusing only on HPV 16 and HPV 18 [22].

In our study, we identified only two statistically significant risk factors associated with HPV detection: use of alcohol increased the odds, and the previous cervical pathology decreased the odds. The association with alcohol use has been previously demonstrated [23, 24], alcohol may contribute to more risky sexual behavior and increased risk of HPV infection. Treatment of previous cancer pathology may remove or alter the infected epithelium, thereby reducing the viral load or eliminating the infected cells, possibly reducing the sensitivity of HPV detection [25]. Several other risk factors in our study were associated with increased, although not statistically significant, odds of HPV detection. Not being married, number of previous pregnancies, and smoking have all been shown to be associated with HPV detection in previous observational studies, although there is big variability among the studies [24]. Association with obstetric history was particularly striking, with women with more than one or more than six pregnancies having more than half reduced odds of HPV detection. Parity has been consistently reported to importantly reduce the risk of HPV infection compared to women without children [24, 26], possibly due to immune system changes during pregnancy [27]. Immunosuppression, especially HIV infection has also been shown to be strongly associated with persistent HPV detection [24, 26], however the HIV prevalence in Kyrgyzstan is very low, estimated at 0.3% by UNAIDS [28].

In our study, only 13% of women with HR-HPV infection were identified with VIA screening. WHO no longer recommends VIA as cervical screening method, due to low and highly variable sensitivity, depending on the capacity and training of the providers [17]. Recently the Kyrgyzstan MOH also aligned the cervical screening guidelines to the WHO [16] recommending HPV testing or cytology as screening tests. However, the challenge for Kyrgyzstan and other resource-limited countries is access to HPV testing, which remains expensive and centralized, and unavailable for many women at risk.

## Limitation and strenghts

One of the limitations of our study was that the population included only those women who responded to the invitation to undergo cervical cancer screening at the participating study sites, and may not be representative of the overall population The risk factor analysis was based on self-reported demographic and behavioral characteristics, which may be prone to reporting and social desirability bias [29]. On the other hand, this is the first study describing prevalence of HR-HPV in Kyrgizstan, providing first information on HR-HPV prevalence and distribution in this country. The study was conducted in routine setting in primary health facilities, thus providing pragmatic information from routine setting at an early stage of HPV screening introduction in the country.

## Conclusion and recommendations

This study represents the first comprehensive data on the prevalence and distribution of HR-HPV types among 30–49 years old women in three primary facilities in Kyrgyzstan. We show prevalence of HR-HPV of 9%, with HPV 16 being most common type identified, followed by other types grouped into P3 and P5. The findings emphasize the importance of tailored public health interventions, including expanded HPV vaccination, and enhancing and scaling HPV-testing based cervical cancer screening programs. Further research is needed to evaluate the impact of these interventions on reducing the burden of HPV-associated cervical cancer in Kyrgyzstan.

## Data Availability

The minimal dataset underlying the findings of this paper are available on request, in accordance with the legal framework set forth by MSF data sharing policy. Readers can contact the MSF generic address data.sharing@msf.org or the corresponding author to request the data that can be shared with researchers subject to the establishment of a data sharing agreement to provide the legal framework for data sharing.

## References

[1] Chan CK, Aimagambetova G, Ukybassova T, Kongrtay K, Azizan A. Human papillomavirus infection and cervical cancer: Epidemiology, Screening, and Vaccination—Review of current perspectives. J Oncol. 2019;2019(1):3257939. 31687023 10.1155/2019/3257939 PMC6811952

[2] World. Human Papillomavirus and Related Diseases, Summary Report 2023 [Internet]. [cited 2025 Jul 25]. Available from: https://hpvcentre.net/statistics/reports/XWX.pdf.

[3] Bray F, Laversanne M, Sung H, Ferlay J, Siegel RL, Soerjomataram I, et al. Global cancer statistics 2022: GLOBOCAN estimates of incidence and mortality worldwide for 36 cancers in 185 countries. CA: A cancer. J Clin. 2024;74(3):229–63. 10.3322/caac.21834 38572751

[4] Singh D, Vignat J, Lorenzoni V, Eslahi M, Ginsburg O, Lauby-Secretan B, et al. Global estimates of incidence and mortality of cervical cancer in 2020: a baseline analysis of the WHO global cervical cancer elimination initiative. Lancet Glob Health. 2023;11(2):e197–206. 36528031 10.1016/S2214-109X(22)00501-0 PMC9848409

[5] de Sanjose S, Quint WG, Alemany L, Geraets DT, Klaustermeier JE, Lloveras B, et al. Human papillomavirus genotype attribution in invasive cervical cancer: a retrospective cross-sectional worldwide study. Lancet Oncol. 2010;11(11):1048–56. 20952254 10.1016/S1470-2045(10)70230-8

[6] Shoja Z, Farahmand M, Hosseini N, Jalilvand S. A Meta-Analysis on human papillomavirus type distribution among women with cervical neoplasia in the WHO Eastern mediterranean region. Intervirology. 2019;62(3–4):101–11. 31527382 10.1159/000502824

[7] Sharipova IP, Musabaev EI, Sadirova SS, Suyarkulova DT, Tashev SE, Akhmedova SK, et al. Prevalence of high-risk human papillomavirus genotypes among women in Uzbekistan, 2021–2023. J Gynecol Oncol. 2025;36(1):e7. 39900340 10.3802/jgo.2025.36.e7 PMC11790988

[8] Babi A, Issa T, Issanov A, Akilzhanova A, Nurgaliyeva K, Abugalieva Z, et al. Prevalence of high-risk human papillomavirus infection among Kazakhstani women attending gynecological outpatient clinics. Int J Infect Dis. 2021;109:8–16. 34111543 10.1016/j.ijid.2021.06.006

[9] Kongrtay K, Kadrlodinova N, Sultankulova F, Batpanova A, Kim Y, Zhumasheva D et al. Prevalence of high-grade HPV types among women in Astana, Kazakhstan (2018–2022). Jcmt. 2023;9(0).

[10] Agorastos T, Chatzistamatiou K, Katsamagkas T, Koliopoulos G, Daponte A, Constantinidis T, et al. Primary screening for cervical cancer based on high-risk human papillomavirus (HPV) detection and HPV 16 and HPV 18 genotyping, in comparison to cytology. PLoS ONE. 2015;10(3):e0119755. 25793281 10.1371/journal.pone.0119755 PMC4368762

[11] Tracht J, Wrenn A, Eltoum IE. Primary HPV testing verification: A retrospective ad-hoc analysis of screening algorithms on women doubly tested for cytology and HPV. Diagn Cytopathol. 2017;45(7):580–6. 28436211 10.1002/dc.23726

[12] Harper DM, DeMars LR. HPV vaccines - A review of the first decade. Gynecol Oncol. 2017;146(1):196–204. 28442134 10.1016/j.ygyno.2017.04.004

[13] Human papillomavirus vaccines. WHO position paper, December 2022 [Internet]. [cited 2025 Oct 3]. Available from: https://www.who.int/publications/i/item/who-wer9750-645-672.

[14] 417-kyrgyzstan-fact. -sheet.pdf [Internet]. [cited 2025 Jul 28]. Available from: https://gco.iarc.who.int/media/globocan/factsheets/populations/417-kyrgyzstan-fact-sheet.pdf.

[15] Davies P, Aluloski I, Arifdjanova D, Brcanski J, Davidzenka A, Durdyeva A, et al. HPV vaccination and cervical cancer screening policies and practices in 18 Countries, territories and entities across Eastern Europe and central Asia. Asian Pac J Cancer Prev. 2023;24(5):1781–8. 37247301 10.31557/APJCP.2023.24.5.1781 PMC10495901

[16] Ministry of Health of the Kyrgyz Republic. Clinical Guidelines for Screening and Treatment of Pre-Cancerous Cervical Lesions. 2023.

[17] WHO guideline for screening and treatment of cervical pre-cancer lesions for cervical cancer prevention, second edition. Geneva: World Health Organization. 2021. Licence: CC BY-NC-SA 3.0. IGO. 34314129

[18] cervical-cancer-kgz. -2021-country-profile-en.pdf [Internet]. [cited 2025 Oct 3]. Available from: https://cdn.who.int/media/docs/default-source/country-profiles/cervical-cancer/cervical-cancer-kgz-2021-country-profile-en.pdf?sfvrsn=f7009c08_33&download=true.

[19] Bruni L, Diaz M, Castellsagué X, Ferrer E, Bosch FX, de Sanjosé S. Cervical human papillomavirus prevalence in 5 continents: meta-analysis of 1 million women with normal cytological findings. J Infect Dis. 2010;202(12):1789–99. 21067372 10.1086/657321

[20] Han S, Lin M, Liu M, Wu S, Guo P, Guo J, et al. Prevalence, trends, and geographic distribution of human papillomavirus infection in Chinese women: a summative analysis of 2,728,321 cases. BMC Med. 2025;23(1):158. 40082952 10.1186/s12916-025-03975-6 PMC11907810

[21] Woromogo SH, Ambounda Ledaga N, Yagata-Moussa FE, Mihindou AS. Uterine cervical neoplasms mass screening at the university hospital centre of Libreville, gabon: associated factors with precancerous and cancerous lesions. PLoS ONE. 2021;16(7):e0255289. 34297784 10.1371/journal.pone.0255289 PMC8301670

[22] González JV, Deluca GD, Liotta DJ, Correa RM, Basiletti JA, Colucci MC, et al. Baseline prevalence and type distribution of human papillomavirus in sexually active non-vaccinated adolescent girls from Argentina. Rev Argent Microbiol. 2021;53(1):11–9. 32788072 10.1016/j.ram.2020.06.004

[23] Oh HY, Kim MK, Seo S, Lee DO, Chung YK, Lim MC, et al. Alcohol consumption and persistent infection of high-risk human papillomavirus. Epidemiol Infect. 2015;143(7):1442–50. 25185457 10.1017/S0950268814002258 PMC9507201

[24] del Pino M, Vorsters A, Joura EA, Doorbar J, Haniszewski M, Gudina IA, et al. Risk factors for human papillomavirus infection and disease: A targeted literature summary. J Med Virol. 2024;96(2):e29420. 38377121 10.1002/jmv.29420

[25] Perkins RB, Wentzensen N, Guido RS, Schiffman M. Cervical cancer screening: A review. JAMA. 2023;330(6):547–58. 37552298 10.1001/jama.2023.13174

[26] Bowden SJ, Doulgeraki T, Bouras E, Markozannes G, Athanasiou A, Grout-Smith H, et al. Risk factors for human papillomavirus infection, cervical intraepithelial neoplasia and cervical cancer: an umbrella review and follow-up Mendelian randomisation studies. BMC Med. 2023;21(1):274. 37501128 10.1186/s12916-023-02965-w PMC10375747

[27] Fu K, Yang X, Zhang M, Yin R. The role of innate immunity triggered by HPV infection in promoting cervical lesions. J Mol Med (Berl). 2025;103(7):739–54. 40411606 10.1007/s00109-025-02553-w

[28] Kyrgyzstan | UNAIDS [Internet]. 2025 [cited 2025 Oct 25]. Available from: https://www.unaids.org/en/regionscountries/countries/kyrgyzstan.

[29] King BM. The influence of social desirability on sexual behavior surveys: A review. Arch Sex Behav. 2022;51(3):1495–501. 35142972 10.1007/s10508-021-02197-0 PMC8917098

